# USING VIRTUAL REALITY IN LONG-TERM CARE TO REDUCE SOCIAL ISOLATION

**DOI:** 10.1093/geroni/igad104.1053

**Published:** 2023-12-21

**Authors:** Lillian Hung, Joey Wong, Mona Upreti, Winnie Kan

**Affiliations:** University of British Columbia, Vancouver, British Columbia, Canada; University of British Columbia, Vancouver, British Columbia, Canada; UBC IDEA lab, Vancouver, British Columbia, Canada; University of British Columbia, Vancouver, British Columbia, Canada

## Abstract

Virtual Reality (VR) has become increasingly accessible for older adults, providing opportunities for interventions that address loneliness and social isolation in long-term care. However, the effectiveness of VR programs can be influenced by various factors, such as the backgrounds, preferences, and capacities of the target population. This qualitative study investigates the acceptability and feasibility of a recreational VR program for social engagement in two Canadian long-term care homes since January 2023. The study involved 20 residents (with various levels of cognitive and physical impairments) who participated in weekly VR group sessions facilitated by staff. Ethnographic observation and video-recorded conversational interviews were conducted with residents during the VR sessions. We also conducted 10 focus groups with 20 staff members. Four patient partners were involved as co-researchers in the team. We performed the thematic analysis with patient partners. We identified three themes: (1) storytelling builds residents’ sense of self, (2) positive emotions persist even when the video is forgotten, and (3) VR empowers resident-resident and staff-resident connections. The findings demonstrate that using VR in long-term care settings is feasible and acceptable for older adults with different cognitive and physical impairments. VR programs have the potential to enhance social engagement and support residents’ personhood as a meaningful activity by improving inclusion, social engagement, comfort, and recognition of their identity. Future research could explore the long-term impact of VR experiences in addressing social isolation and loneliness among older adults in long-term care.

